# Case of Enteritis, Terminating in Serous Effusion into the Abdomen; with the Appearances on Dissection

**Published:** 1818-10-01

**Authors:** Archibald Robertson

**Affiliations:** Member of the Royal Medical Society of Edinburgh, &c.


### *Case of Enteritis, terminating in serous Effusion into the Abdomen; with the Appearances on Dissection.*

By Archibald Robertson, M. D. Member of the Royal Medical Society of Edinburgh, &c.

B. T. (a Negro, a native of the island of Madagascar, about 27 or 28 years of age, tall and rather slender,) came to me on the evening of the 19th of May (1814), complaining of severe pain in his abdomen, which he had first felt that same afternoon. He had had no stool from the morning of the day before. On examining his belly, I found it hard, swollen, and tense. Skin hot, tongue chalky, face anxious, p. 96, but by no means strong.

This man was ill of a pleuritic affection, from the 16th to the 26th of last March, and was again confined from the 1st to the 20th of April with cough, and a number of anomalous and uneasy feelings, of which the most prominent was general debility. Upon the whole, he had been pining and gradually losing flesh for several months before: this I attributed partly to the venesections and other evacuations which he had necessarily undergone during his pectoral attack; partly to the severity of that attack itself; and partly, perhaps, to an uncommon sullenness and discontentedness of temper, by which he was distinguished much more than even Negroes usually are.

His present ailment being obviously an attack of Enteritis, I determined to treat it vigorously; as in warm climates, such as where we then were, the progress and termination of all diseases of increased action are more rapidly and more frequently fatal than in temperate regions. I therefore bled him to as considerable an extent as his strength would permit, put him to bed, administered ʒjß of the ol. Ricini, and applied a bladder half full of warm water to his abdomen.

By eleven o'clock of the same evening, he had two pretty copious dejections, but without any relief of abdominal pain: umbillicus still painful on pressure; some vomiting. Blood was again drawn, to the extent of ℥xxv.

I ought to have mentioned before, that a very small hard tumour was felt on the linea alba, half way between the stomach and navel. This, though it a good deal resembled some hard foreign body lodged in the lower arch of the stomach, or in the convolution of some intestine, I clearly ascertained to be a ventral hernia: it could be easily pushed inside the abdominal parietes, and did not protrude again till forced out by coughing, or some other accidental motion.

May 20th (second day of illness), p. 78, skin more cool, abdomen less tense; but pain in it still continues, and is increased by pressure. Venesection was again performed, but he became faint after losing ℥xvj. This was followed by an entire abatement of the pain, which never afterwards returned. For the three following days, he took no medicines, except a single dose of ol. Ricini; for diet he had rice, of which he was very fond.

On the 23d of May, his belly and legs began to swell: he had no pain in the former. His pulse and temperature were natural; and the prominent symptoms were those of ascites. Laxatives and the other usual hydragogues were now prescribed, and a generous diet allowed; but the hydropic symptoms increased, while his general strength daily diminished.

On the 29th of May, he was attacked with violent diarrhœa from some unknown cause; though this was arrested in three days, yet it pulled him down prodigiously. On the 1st of June, he was free from diarrhœa, but his belly was now greatly distended. On the 22d, he was seized with dyspnœa, and expired the same night. Cordials of various sorts had been freely given during the last two days of his life.

### DISSECTION.

On dividing the linea alba, I found a ventral hernia (as above mentioned), but without any marks of being either remotely or recently strangulated. On cutting the peritoneum lining the abdominal parietes, I found a large collection of green serous fluid; the quantity could not be less than two gallons!

The intestines were so absolutely deformed, that it was not till after a minute or two, and comparing the relative situation of the parts, that I was able to tell the different portions of the canal. All the intestinal convolutions were streaked with the ramifications of minute red vessels (usually called blood-shot), indicating high previous inflammation. The omentum had a singular appearance: it was thick, hard, glistening and knotty, and looked like a confused mass of diseased cartilage, binding together the stomach and transverse arch of the colon. It reached no farther than the colon, and did not hang over, in front of the other intestines, like an apron.

All the intestines were diseased; their coats being thickened, and their calibre contracted. The colon particularly was most diseased in the latter way: I could hardly introduce two fingers along its transverse arch. The inner surface of all the intestines seemed healthy enough.

How shall I describe the mesentery? Instead of being a thin membrane, it had become a solid semi-diaphanous mass, about an inch and a half or two inches in thickness! It more nearly resembled the thick fatty parts of a boiled turtle than any thing else that suggests itself to my imagination. The mesenteric glands were very large and hard.

The fundus of the bladder was externally *blood-shot*, like the intestines. When cut into, I found its coats nearly half an inch thick. The spleen was thrice its natural size, and when gently squeezed, it yielded under the pressure; pieces of it could be easily torn away, leaving a granular irregular fracture, as if it had been quite disorganized and decayed. The pancreas was immensely enlarged and indurated, as was also the liver.

In fact, not a single organ in the abdomen (the stomach and kidnies excepted) but was strangely and monstrously altered by organic disease. Never did I see such a dissection!

In the thorax, the lungs, &c. were healthy. The heart was uncommonly small, but this did not appear to be the effect of morbid action, for its structure was sound. Nature is known to make a difference in the size of this organ, as well as of other parts of the body. For instance, it was found on dissection, that the immortal Nelson had one of the smallest hearts ever seen in the human species; yet he never was subject to cardiac or other pectoral complaints. I state this fact with regard to his Lordship, on the authority of an interesting narrative published by Dr. Beattie, physician of the fleet, and surgeon to his Lordship's flag at the battle of Trafalgar.

### REMARKS.

How is the immediate cause of this man's death to be explained? His debility, his loss of flesh, and his "pining atrophy," for several previous months, are more than sufficiently accounted for by the appearances on dissection; yet they afford no reason why he might not have lingered out longer. It is highly probable, nay certain, I may say, that, from the evacuations he underwent for enteritis, the balance of the circulation was overthrown; the capillary and exhalent systems lost all their tone, readily yielded to the impulse *a tergo*, and allowed the serous part of the blood to escape at the point where these delicate vessels were rendered weakest by previous organic lesion. In this way, as the effusion was rapid, it would, in some measure, have similar effects to internal hæmorrhage.

The termination of enteritis in serous effusion is, I believe, a rare occurrence; in this view, the case seems not altogether destitute of interest. But besides, the appearances on dissection must afford ample scope for reflection to the pathological physiologist, as they furnish a proof how long life may be protracted under even extensive and numerous visceral organic changes, any one of which might be presumed capable of speedily putting a period to its farther existence.

*September*, 1818.

